# Inland post-glacial dispersal in East Asia revealed by mitochondrial haplogroup M9a'b

**DOI:** 10.1186/1741-7007-9-2

**Published:** 2011-01-10

**Authors:** Min-Sheng Peng, Malliya Gounder Palanichamy, Yong-Gang Yao, Bikash Mitra, Yao-Ting Cheng, Mian Zhao, Jia Liu, Hua-Wei Wang, Hui Pan, Wen-Zhi Wang, A-Mei Zhang, Wen Zhang, Dong Wang, Yang Zou, Yang Yang, Tapas Kumar Chaudhuri, Qing-Peng Kong, Ya-Ping Zhang

**Affiliations:** 1 State Key Laboratory of Genetic Resources and Evolution, Kunming Institute of Zoology, Chinese Academy of Sciences, Kunming, China; 2 KIZ/CUHK Joint Laboratory of Bioresources and Molecular Research in Common Diseases, Kunming, China; 3 Laboratory for Conservation and Utilization of Bio-resources, Yunnan University, Kunming, China; 4 Key Laboratory of Animal Models and Human Disease Mechanisms of the Chinese Academy of Sciences & Yunnan Province, Kunming Institute of Zoology, Kunming, China; 5 Cellular Immunology Laboratory, Department of Zoology, University of North Bengal, Siliguri, India; 6 Graduate School of the Chinese Academy of Sciences, Beijing, China

## Abstract

**Background:**

Archaeological studies have revealed a series of cultural changes around the Last Glacial Maximum in East Asia; whether these changes left any signatures in the gene pool of East Asians remains poorly indicated. To achieve deeper insights into the demographic history of modern humans in East Asia around the Last Glacial Maximum, we extensively analyzed mitochondrial DNA haplogroup M9a'b, a specific haplogroup that was suggested to have some potential for tracing the migration around the Last Glacial Maximum in East Eurasia.

**Results:**

A total of 837 M9a'b mitochondrial DNAs (583 from the literature, while the remaining 254 were newly collected in this study) pinpointed from over 28,000 subjects residing across East Eurasia were studied here. Fifty-nine representative samples were further selected for total mitochondrial DNA sequencing so we could better understand the phylogeny within M9a'b. Based on the updated phylogeny, an extensive phylogeographic analysis was carried out to reveal the differentiation of haplogroup M9a'b and to reconstruct the dispersal histories.

**Conclusions:**

Our results indicated that southern China and/or Southeast Asia likely served as the source of some post-Last Glacial Maximum dispersal(s). The detailed dissection of haplogroup M9a'b revealed the existence of an inland dispersal in mainland East Asia during the post-glacial period. It was this dispersal that expanded not only to western China but also to northeast India and the south Himalaya region. A similar phylogeographic distribution pattern was also observed for haplogroup F1c, thus substantiating our proposition. This inland post-glacial dispersal was in agreement with the spread of the Mesolithic culture originating in South China and northern Vietnam.

## Background

The climatic oscillation and the related ecological changes around the Last Glacial Maximum (LGM; approximately 26.5 to 19 kilo-years ago (kya)) [1] were suggested to exert substantial influence on prehistoric migrations and demographic changes in modern humans [2]. In East Asia, archaeological studies have indicated that great changes occurred in the wake of the LGM [3,4]. For instance, the microblade technology appeared and became popular during the LGM in northern China [5]; some early settlements were abandoned [6] and people probably moved to the south due to the deteriorating environmental conditions [7]. After the LGM, improved climate allowed humans to re-colonize the high latitude regions [8]. However, whether the ancient dispersals around the LGM left any detectable genetic footprints in the gene pool of the contemporary East Asians was still elusive.

In the past decades, genetic data of mitochondrial DNA (mtDNA) and the non-recombining region of Y-chromosome (NRY) have been widely employed to reconstruct human prehistory [9,10]. In Europe, the detailed phylogeographic dissection of matrilineal pools has discerned some haplogroups as the candidate markers for tracing the dispersal(s) after the LGM, which could be assigned as the Late Glacial (before the Holocene) and the post-glacial (after the Younger Dryas but before the Neolithic) re-colonization, respectively [11]. Recently, this strategy has also been applied to other regions (for example, West Asia [12], South Asia [13], and Southeast Asia [14]), yielding many valuable insights into the prehistoric demographic events around the LGM.

To trace the ancient dispersal of modern humans in East Asia around the LGM, we carried out a detailed phylogeographic analysis on a high resolution mtDNA marker. We focused our attention particularly on East Eurasian specific mtDNA haplogroup M9a'b for four reasons: 1) M9a'b distributes widely in mainland East Asia [14] and is relatively concentrated in Tibet (approximately 19.2%) [15,16] and its surrounding regions, including Nepal (approximately 11.6%) [17], Sikkim (approximately 11.7%) [18] and northeast India (approximately 8.6%) [18,19]. 2) The phylogeny of haplogroup M9a'b indicated that this clade might be involved in some northward migrations into East Asia from Southeast Asia [14]. 3) The coalescent time estimates of certain sub-haplogroups of M9a'b, for example, M9a (approximately 12 to 15 kya) [14,16] and M9d (approximately 12 kya) [16], suggested that these lineages were likely associated with some post-LGM dispersal(s) in East Asia [14], especially in Tibet [15,16]. 4) In addition to its high frequency, the relatively high genetic diversity, as revealed by the mtDNA control region hypervariable segment I (HVS-I) information in Tibet [20], suggested that Tibet might serve as the potential differentiation center of M9a'b sub-haplogroups. All these lines of evidence appeared to imply that Tibet might be a candidate source for the post-LGM dispersal in East Asia. Together, the detailed dissection of haplogroup M9a'b would provide insightful information for the ancient movement of modern humans in East Asia around the LGM.

## Results

### M9a'b phylogenetic tree based on mtDNA genome information

**Figure 1:**
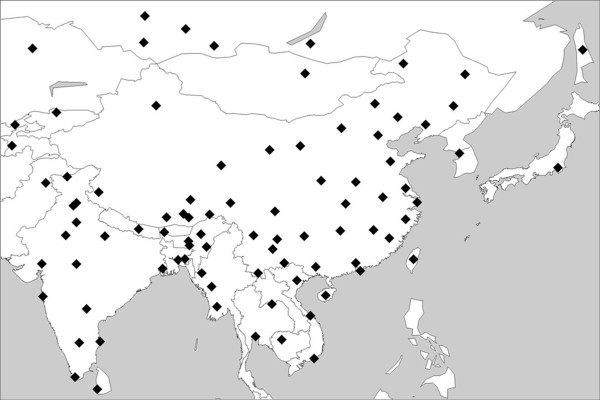
**Geographic locations of populations surveyed in this study**. For more details regarding the populations, refer to Additional file 2.

**Figure 2:**
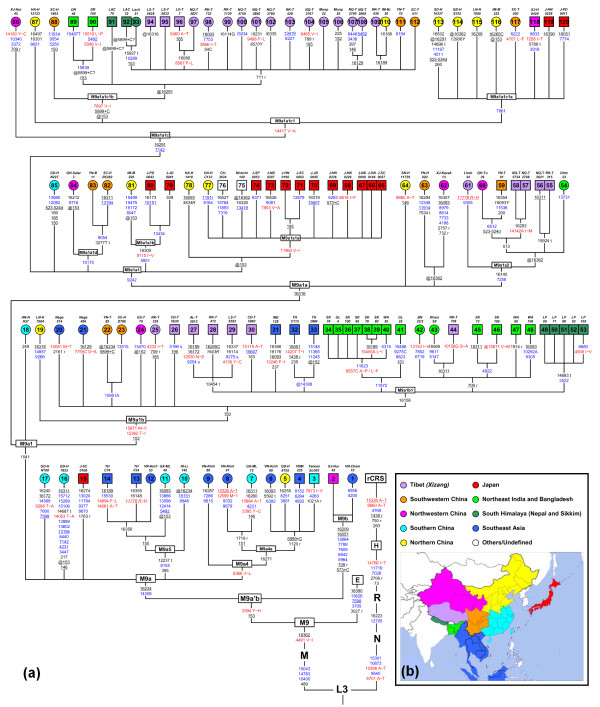
**Classification tree of M9a'b rooted in haplogroup M9**. **(a) **The tree includes 120 complete sequences and illustrates sub-haplogroup affiliations (see Additional file 4). Sequences 1, 5 to 12, 16 to 25, 42 to 43, 54, 60 to 64, 77 to 78, 81 to 88, 93, 97 to 117 were newly collected and indicated as circles, while the others from published sources were represented as squares. The nucleotide positions in the sequences were scored relative to the revised Cambridge Reference Sequence (rCRS) [52]. Transitions are shown on the branches and transversions are further annotated by adding suffixes. The deletions and insertions are demonstrated by ''d'' and ''+'', respectively. Amino acid replacements are in red and marked by a single-letter code, whereas synonymous replacements are in blue. Changes in transfer RNA and ribosomal RNA genes are denoted by "t" and "r", respectively. The prefix @ designates back mutation and recurrent variants are underlined. "R" and "Y" specify the heteroplasmic status of A/G and C/T at a certain site, respectively. All heteroplasmic variants and the potential pathogenic transition 11778 [62] are not considered in the ages estimates and are marked in italics. The insertion of C at site 5899 seemed to be missing in sequences 88 to 91, which is tentatively noted as "@5899+C?". **(b) **The geographic origin of samples is shown by different colors corresponding to their respective different locations in the map.

After incorporating the 59 newly sequenced mtDNA genomes, the phylogeny of haplogroup M9a'b was greatly improved in the context of East Eurasians (Figure 1). The overall structure of the tree turned out to be much more complex than we had ever thought [14-16,18,20,21] (Figure 2). For instance, a number of basal lineages branched directly from the M9a'b root and shared merely two variants 16234 and 14308 with previously defined haplogroups M9a and M9d [16,18]. To update the definitions of haplogroup M9a'b and its sub-haplogroups and to avoid potential confusion, we kept the definition of M9a'b, but expanded that of M9a (now defined by transitions 14308 and 16234) to embrace M9a1 (defined by variant 1041), M9a4 (defined by transition 6366), and M9a5 (determined by variants 385, 8155, and 12237). Nomenclature of some other sub-haplogroups, such as M9a1b (former M9d [16,18]), M9a1a1 (former M9a [18]), and M9a1a2 (former M9e [16]), were adjusted accordingly (Figure 2). It should be mentioned that although the validity of M9a1a might be questionable because this haplogroup was defined solely by a control region variation at site 16316, its two major clades (M9a1a1 and M9a1a2) were determined by additional coding region variants. The updated nomenclature has been deposited to PhyloTree (http://www.phylotree.org, mtDNA tree Build 10) [22].

Based on the updated M9a'b phylogeny, some interesting features could be discerned. With the exception of M9a1, most basal branches of M9a were distributed in southern China (6/15) and Southeast Asia (7/15); this pattern suggested that M9a might have a southern origin. The distribution pattern of M9a1 was rather complex: although this haplogroup did bear some genetic imprints of southern origin by harboring a basal lineage (that is, *HN-H H27*) from southern China, its effect had actually extended to northern China and Japan (for example, M9a1a1a, M9a1a1b, and M9a1a1c1a), as well as, western China (that is, southwestern China, northwestern China, and Tibet), northeast India (including Bangladesh), and the south Himalaya region (for example, M9a1b1, M9a1a2, and M9a1a1c1b). Based on this pattern, it seemed that haplogroup M9a1 had most likely been involved in some northward and westward dispersal(s) in East Asia.

### Phylogeographic distribution

**Figure 3:**
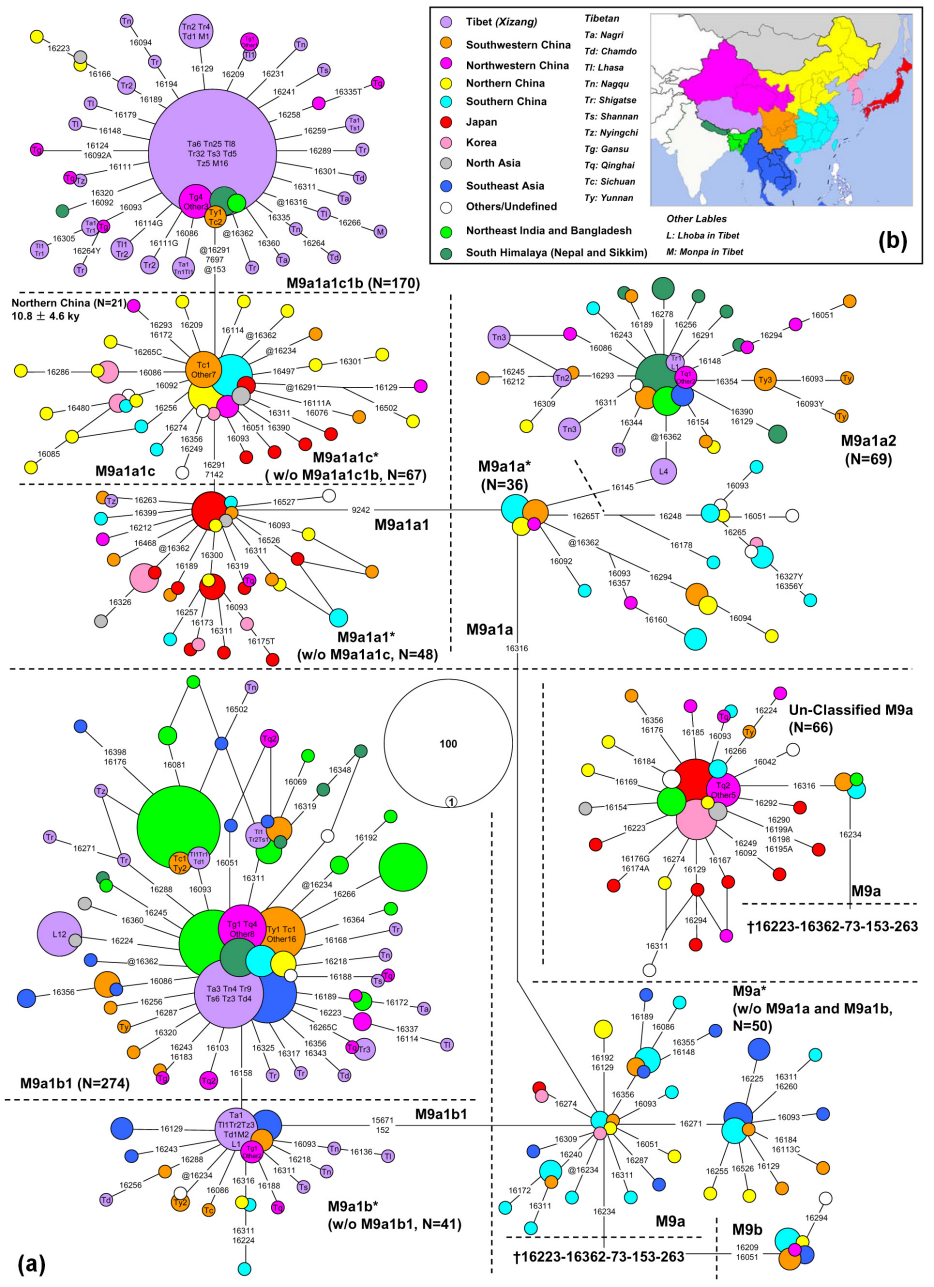
**Median-joining network of HVS-I haplotypes observed in 837 M9a'b mtDNAs**. **(a) **mtDNA control region variations and/or certain coding region sites were considered to improve the resolution of the median-joining network. The variants are transitions, and transversions are further highlighted by adding suffixes A, C, G and T. "Y" means heteroplasmic status C/T, and "@" means a back mutation. The "†" labels the putatively ancestral node of haplogroup M9a'b. **(b) **The geographic origin of samples is shown by different colors corresponding to their respective locations on the map. For the samples from Tibet, the related population information is also noted.

The updated phylogenetic tree of haplogroup M9a'b provided a basis for us to reanalyze the previously published data and to perform a well-defined phylogeographic analysis of this haplogroup. To better characterize the demographic history of M9a'b, the median-joining network was constructed based on all available M9a'b mtDNAs (Figure 3). In general, the network (based on the combined information of the control region and partial coding region) was in agreement with the phylogeny of the entire mtDNA genomes (Figure 2). Our comprehensive study of haplogroup M9a'b substantiated the notion that the origin of this haplogroup was most likely located in southern China and/or mainland Southeast Asia. As displayed in Figure 3, most of the basal lineages within M9a (that is, M9a*; excluding M9a1a and M9a1b mtDNAs) came from southern China, southwestern China, and Southeast Asia, strongly suggesting a southern origin of M9a. This result received further support from M9b: 9 of the 11 M9b sequences were observed in southern China, southwestern China, and Southeast Asia, while the remaining two were found in northwestern China and northern China, respectively (Figure 3; see Additional file 1).

**Figure 4:**
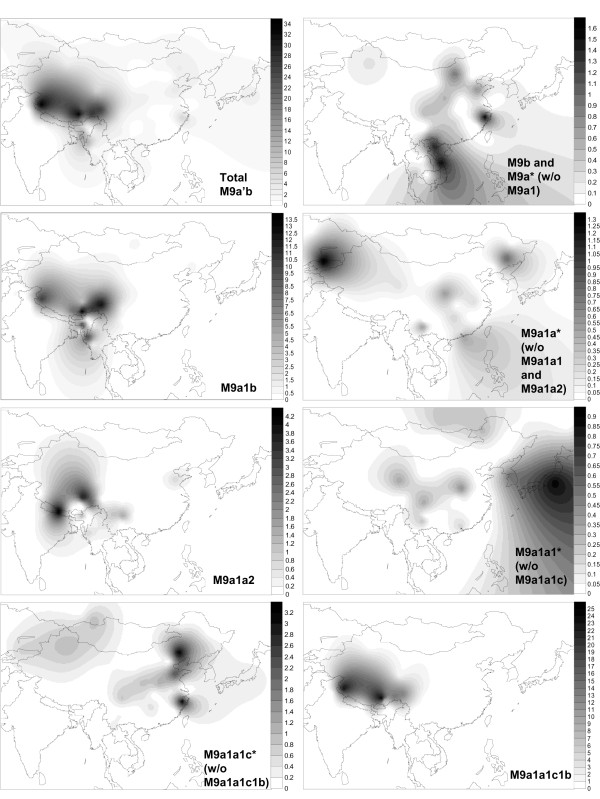
**Spatial frequency distributions of haplogroup M9a'b and its sub-haplogroups**. Populations and corresponding frequency values are listed in Additional file 2. Fifty mtDNAs were not included in computing the population frequency because the essential information was missing or not reported in the original studies (see Additional file 1). The spatial-frequency distributions were created using the Kriging algorithm of the Surfer 8.0 package.

Similar to the observation from the phylogenetic tree of the complete mtDNAs (Figure 2), the median-network showed that the dominant clade (M9a1) within M9a presented a quite different geographic distribution pattern from its sister cluster M9a* (Figure 3). Within haplogroup M9a1b, the basal lineages were mainly restricted to western China and Myanmar, whereas M9a1b1 spread not only in western China and Myanmar, but also in northeast India and the south Himalaya region (Figure 3). The basal lineages belonging to M9a1a* were mainly found in southern China (Figure 3). One of its derivatives, haplogroup M9a1a2, displayed a restricted distribution in western China, Myanmar, northeast India, and the south Himalaya region (Figure 3 and 4; see Additional file 2), and presented a similar pattern to that of haplogroup M9a1b1. Nevertheless, haplogroup M9a1a1 showed a distinct distribution pattern: most of M9a1a1 basal lineages were distributed in southern China, southwestern China, as well as, northern China, Japan and Korea, whereas its major sub-haplogroup M9a1a1c was prevalent in northern China, Korea, and Japan (Figure 3 and 4; see Additional file 2). Remarkably, the M9a1a1 lineages found in Tibet were almost clustered into haplogroup M9a1a1c1b.

### Coalescence age estimates

The large number of M9a'b samples with complete mtDNA genome information, as well as the network with a high-resolution, allowed us to estimate the coalescence ages of the nodes (*viz*., ancestral haplotypes within M9a'b) of interest. Although there were some exceptions, the estimated ages based on different calibrated rates were in general accordance with each other and seemed to be quite robust (Table 1). The whole haplogroup M9a'b showed a coalescence time of approximately 26 to 28 kya. The estimated coalescence age of haplogroup M9a was approximately 18 to 23 kya. Within haplogroup M9a1, haplogroups M9a1a1 and M9a1b1 emerged around 14 to 17 kya and 9 to 12 kya, respectively. For haplogroup M9a1a2, because of the small number of available mtDNA genome sequences, which would bias the age estimates, we adopted the age estimation result based on HVS-I data (11.3 ± 3.5 kya). As a result, nearly all the age estimates placed the origin of haplogroup M9a1a1 in the Late Glacial episode, whereas haplogroups M9a1b1 and M9a1a2 are in a more recent post-glacial period (the end of the Pleistocene and the early Holocene), despite a fact that these ages should be received with caution [23,24].

**Table 1:** Estimated coalescence ages of mtDNA haplogroup M9a'b and its sub-haplogroups based on different calibration rates.

		Entire mitochondrial genome				Only synonymous mutations					Transitions in 16090 to 16365			
**Node/Clade**	**N**^**a**^	**ρ**	**σ**	**T**^**b **^**(ky)**	**ΔT**^**b **^**(ky)**	**ρ**	**σ**	**T**^**c **^**(ky)**	**ΔT**^**c **^**(ky)**	**N**^**a**^	**ρ**	**σ**	**T**^**d **^**(ky)**	**ΔT**^**d **^**(ky)**

M9a'b	120	10.83	2.01	28.0	5.2	3.28	1.14	26.2	9.1					
M9a	118	8.86	1.48	22.9	3.8	2.23	0.59	17.8	4.7					
M9a* (w/o M9a1)	15	7.40	1.11	19.1	2.9	2.73	0.52	21.8	4.2	50	1.20	0.46	22.6	8.7
M9a5	5	4.40	1.26	11.4	3.3	1.40	0.53	11.2	4.2					
M9a4	7	5.57	1.32	14.4	3.4	1.71	0.49	13.7	4.0					
M9a1	103	8.07	1.36	20.9	3.5	2.16	0.67	17.2	5.3					
M9a1b	35	5.03	1.35	13.0	3.5	1.37	0.37	11.0	3.0					
M9a1b* (w/o M9a1b1)	8	3.13	1.04	8.1	2.7	0.88	0.38	7.0	3.0	41	0.51	0.16	9.7	3.0
M9a1b1	27	3.59	0.68	9.3	1.8	1.52	0.47	12.1	3.8	274	0.55	0.23	10.5	4.3
M9a1a	67	7.19	1.43	18.6	3.7	2.60	1.01	20.8	8.1					
M9a1a2	8	3.13	1.17	8.1	3.0	0.63	0.33	5.0	2.6	65^e^	0.60	0.19	11.3	3.5
M9a1a1	56	6.59	1.37	17.0	3.5	1.77	0.66	14.1	5.3					
M9a1a1* (w/o M9a1a1c)	21	4.00	1.01	10.3	2.6	1.43	0.32	11.4	2.6	48	0.77	0.30	14.5	5.7
M9a1a1c	35	6.14	1.55	15.9	4.0	0.97	0.28	7.8	2.2					
M9a1a1c1	34	5.21	1.25	13.5	3.2	0.94	0.28	7.5	2.3					
M9a1a1c1* (w/o M9a1a1c1b)	9	3.33	1.03	8.6	2.7	1.67	0.94	13.3	7.5	67	0.39	0.14	7.3	2.6
M9a1a1c1b	25	3.88	1.32	10.0	3.4	0.68	0.18	5.4	1.5					
M9a1a1c1b (@16291)	24	2.88	0.94	7.4	2.4	0.58	0.18	4.7	1.4	170	0.32	0.08	6.1	1.5
M9a1a1c1b (@16291-711)	21	1.90	0.35	4.9	0.9	0.48	0.16	3.8	1.3					

^a ^Number of mtDNA sequences.

^b ^Using the corrected molecular clock proposed by Soares *et al*. [60].

^c ^According to the recalibrated synonymous rate of Loogväli *et al*. [61]. The rate of Soares *et al*. [60] (7,884 years/synonymous substitution) was similar to Loogväli *et al*. [61] (7990 years/synonymous substantiation), and age estimates based on this rate were not listed in the Table.

^d ^Using the corrected molecular clock proposed by Soares *et al*. [60].

^e ^The numbers of mtDNAs refer the sequences allocated into M9a1a2 with additional back-mutation 16362.

## Discussion

**Figure 5:**
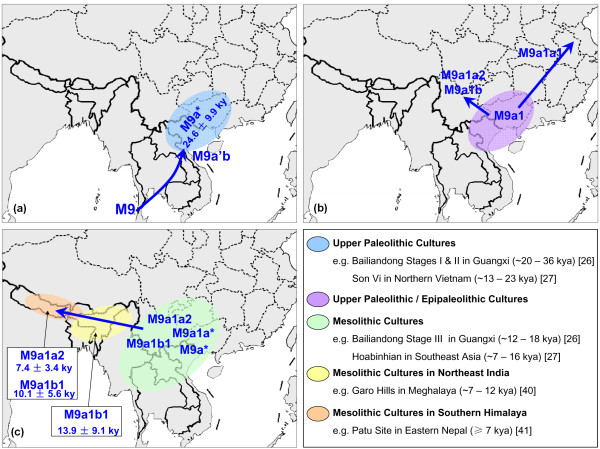
**The putative migratory routes of M9a'b and the distribution of the potentially associated archaeological evidence**. Arrows refer to the dispersal direction but do not denote precisely defined geographic routes. The ages of specific haplogroups were based on the mtDNA control region sequences: **(a) **M9a* lineages in southern China and Southeast Asia; **(b) **M9a1 in southern China; and **(c) **M9a1b1 lineages in northeast India, and M9a1b1 and M9a1a2 lineages in the south Himalaya region.

Although some previous studies based on limited information from mtDNA control region suggested that haplogroup M9a'b might trace its origin in North Asia/northern China [16,25] or Central Asia (including Tibet) [20], evidence from entire mtDNA genomes and extensive phylogeographic analyses unanimously indicated that this haplogroup was originated in southern China and/or Southeast Asia, a vast region containing contemporary northern Vietnam and South China (that is, Guangxi, Guangdong, and Hainan). This result was consistent with the previous observation on haplogroup E, the sister clade of M9a'b [14], and thus provided further evidence in support of the common origin of haplogroup M9 (embracing M9a'b and E) in Southeast Asia [14]. Moreover, the emergence of M9a'b and/or M9a and their related early dispersal in southern China and/or Southeast Asia (Figure 4) around 18 to 28 kya (Table 1) was in agreement with the rise of the Upper Paleolithic culture within this region (Figure 5a). During this period, the first (approximately 26 to 36 kya) and second (approximately 20 to 26 kya) stages of Bailiandong culture in Guangxi [26] and the Son Vi culture (also Sonviian; approximately 13 to 23 kya) in northern Vietnam [27] appeared and showed tight links (for example, cobble choppers and blades) with each other [26].

Our phylogeographic analysis of haplogroup M9a'b further revealed some distinct distribution patterns of its sub-haplogroups. In particular, M9a1b and M9a1a2 showed a restricted distribution in western China, Myanmar, northeast India, and the south Himalaya region (Figure 4; see Additional file 2), but were virtually very rare or absent in northern China and Northeast Asia and even southern China (the suggested place of origin of M9a'b and M9a1), indicating that both haplogroups might have distinct origins from the other M9a'b sub-haplogroups. Meanwhile, M9a1a2 and M9a1b coincidentally shared a similar expansion age (approximately 9 to 12 kya; Table 1), which indicated that both haplogroups might have been involved in the same demographic event. Together, the current distribution pattern of haplogroups M9a1b and M9a1a2 was likely attributed to an inland post-glacial dispersal event, which started from southern China along with the differentiation of M9a1 (approximately 17 to 21 kya; Table 1; Figure 5b), then moved westward to western China, and finally to northeast India and the south Himalaya region (Figure 5c). Nevertheless, the phylogeographic pattern of M9a1a1 suggested some northward Late Glacial dispersal(s). In particular, the enrichment of haplogroup M9a1a1c1b in Tibet was likely to be explained by some recent local expansions, such as the Neolithic expansion [28,29] in this region.

**Figure 6:**
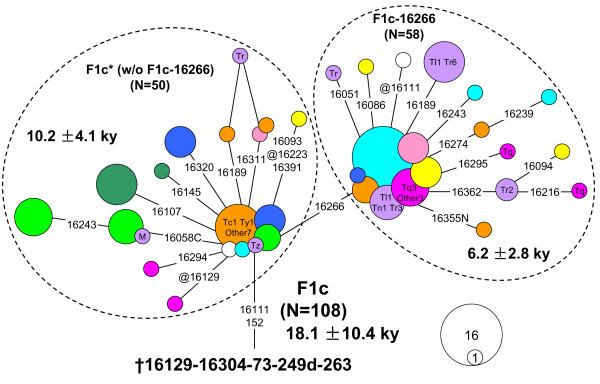
**Median-joining network of HVS-I haplotypes observed in 108 F1c mtDNAs**. All sequences were retrieved from the published data (see Additional file 3). For the information of the labels, see Figure 3 and its legend.

It is possible that the observed pattern based on a single haplogroup (that is, M9a'b) might be biased by genetic drift, natural selection, and later population dynamic events [30]. So we tried to look for the parallel genetic evidence from the published data: haplogroup F1c [31,32], with mtDNA control region motif as 16111-16129-16304-152-249d, was found to show a similar phylogeographic pattern with haplogroup M9a1. As previous studies had indicated that haplogroups F1 and F1a (a sister clade of F1c) probably had an origin in southern China and/or Southeast Asia [32-34], haplogroup F1c also probably originated in the same region. The network based on 108 mtDNA control region sequences (see Additional file 3) suggested that several branches derived directly from the root type of F1c, and these lineages were mainly restricted in western China, northeast India, and the south Himalaya region (Figure 6). Regardless of the major branch defined by variant 16266, the expansion time of haplogroup (paragroup) F1c* was estimated to be 10.2 ± 4.1 kya. Therefore, the differentiation of haplogroup F1c* had likely witnessed certain inland post-glacial dispersal from southern China and/or southwestern China to northeast India and the south Himalaya region, which mirrored the distribution pattern of M9a1b and M9a1a2.

The proper interpretation of the obtained genetic data to reconstruct complex colonization scenarios would benefit from the incorporation of archaeological materials. After the LGM, around 12 to 15 kya, great cultural changes in South China and northern Vietnam were suggested to be associated with the prevalence of the Mesolithic culture, such as the Hoabinhian culture [27,35] and the third stage of Bailiandong culture [26,36]. The expansions of these Mesolithic cultures in southern China and Southeast Asia were already discussed in some recent studies [37,38]. Intriguingly, the timing for our proposed inland post-glacial dispersal scenario was largely overlapped with the Mesolithic period, and more importantly, this inland route from southwestern China to northeast India and the south Himalaya region was in coincidence with the Hoabinhian links connecting southwestern China [39], northeast India [40], and Nepal [41,42] (Figure 5c). It seemed that the advanced technology (for example, pottery [26,36,43]) and the improved climate would be the major factors in triggering the post-glacial dispersal. However, other factors such as the dispersal of language groups and the expansion of agriculture could not be neglected completely. Considering some major branches within M9a'b were relatively concentrated in different Tibeto-Burman and Khasi-Khmuic populations (see Additional file 1), the dispersals of Tibeto-Burman [44] and Austro-Asiatic populations [45], together with the intergroup genetic admixture [45], were likely to shape the current distribution pattern of M9a'b. Further work on more genetic markers (for example, NRY, genome-wide single nucleotide polymorphisms, and even ancient DNA) with extensive sampling will be required to further confirm our speculation regarding the prehistoric peopling scenario(s) in East Asia.

## Conclusions

Our comprehensive phylogeographic analyses of mtDNA haplogroup M9a'b revealed that southern China and/or Southeast Asia served as a source of the post-LGM dispersal in East Asia. Most importantly, our results provided the first direct genetic evidence in support of the existence of an inland dispersal in mainland East Asia from southern China, through western China, to northeast India and the south Himalaya region. This dispersal was likely triggered by the improved climate and the advanced Mesolithic culture, and had played important roles in shaping the matrilineal gene pool of modern East Asians.

## Methods

### Subjects

A total of 837 candidate M9a'b mtDNA samples (583 from the literature and 254 from this study; see Additional file 1), with specific mtDNA control region motif 16223-16234-16362-153 and/or coding region diagnostic site 3394 or 4491, were pinpointed from over 28,000 subjects residing across East Eurasia (Figure 1; see Additional file 2). All subjects recruited in this study were interviewed with informed consent to ascertain their ethnic affiliations. To better understand the phylogeny within M9a'b, besides the 61 published M9a'b mtDNA genome sequences that were retrieved from the literature and GenBank (see Additional file 4), an additional 59 representatives were selected from our own samples for complete mtDNA sequencing, with a special attempt to cover the widest range of internal variation within the haplogroup [46]. By virtue of the updated phylogeny of haplogroup M9a'b, we further classified the remaining M9a'b candidates based on the specific coding region motifs (for our own samples; see Additional file 1) and/or by matching and near-matching [32,47] with the well-defined M9a'b lineages (for the reported mtDNAs from the literature). Using this strategy, the vast majority of the M9a'b mtDNA samples (771/837) could be unambiguously allocated into specific sub-haplogroups within M9a'b, whereas the remaining 66 sequences (all from the literature) could only be roughly assigned into M9a'b* due to lack of further information (see Additional file 1).

### Sequence analysis

The sequencing protocol and phylogeny reconstruction were performed as fully described before [48,49], and some caveats for data quality-control were followed during the data generation and handling [50,51]. Sequences were edited and aligned by using Lasergene (DNAStar Inc., Madison, Wisconsin, USA) and variations were scored relative to the revised Cambridge Reference Sequence (rCRS) [52]. For the C-stretch length variants in the control region, we followed the rules proposed by Bandelt and Parson [53]. The transition at 16519 and the C-length polymorphisms in regions 16180 to 16193 and 303 to 315 were disregarded in the analyses. The classification of the variants of each mtDNA genomes was performed with mtDNA GeneSyn 1.0 http://www.ipatimup.pt/downloads/mtDNAGeneSyn.zip[54] and MitoTool http://mitotool.org/index.html[55]. Sequences generated in this study have been deposited in GenBank (Accession Nos. GQ337542, GQ337575, GQ337588, and HM346881 to HM346936).

### Phylogenetic tree construction and data analysis

The phylogenetic tree of 120 M9a'b complete mtDNA sequences was reconstructed manually and checked by NETWORK 4.516 http://www.fluxus-engineering.com/sharenet.htm. For the HVS data and/or partial coding region, the median-joining network of 837 M9a'b mtDNA sequences was constructed manually and was further checked by using the Network 4.516 [56]. The counter maps of spatial frequencies [57] were constructed to elaborate the geographic distribution patterns of haplogroup M9a'b and its sub-haplogroups using the Kriging algorithm of Surfer 8.0 (Golden Software Inc. Golden, Colorado, USA).

The average sequence divergence (ρ) of the haplotypes to their most recent common ancestor, accompanied by a heuristic estimate of the standard error (σ), was calculated as fully described before [58,59]. Then, the ρ ± σ value was converted into the coalescent age for certain haplogroup by using the most recently proposed calibration rates for mtDNA mutations [60] and only synonymous substitutions [61], respectively. For the control region, we adopted the rate of 18,845 years per transition between 16090 and 16365 [60].

## Abbreviations

HVS: hypervariable segment; kya: kilo-years ago; LGM: Last Glacial Maximum; mtDNA: mitochondrial DNA; NRY: non-recombining region of Y-chromosome; rCRS: revised Cambridge Reference Sequence.

## Authors' contributions

MSP, MGP, BM, YTC, MZ, JL, HWW, HP, WZW, AMZ, WZ, DW, YZ, YY, and TKC performed the experiments. MSP, YGY, and QPK analyzed the data. MSP, QPK, and YPZ conceived and designed the experiments. MSP, YGY, QPK, and YPZ wrote the paper. All authors read and approved the final manuscript.

## Supplementary Material

Additional file 1 **List of M9a'b mtDNAs identified in East Eurasians**. This is an EXCEL file describing the information of 837 M9a'b mtDNAs. Click here for file

Additional file 2 **Population distribution of haplogroup M9a'b**. This is an EXCEL file providing information includes sample sizes, geographic locations and distributions of sub-haplogroups. Click here for file

Additional file 3 **List of F1c lineages identified from the published data**. This is an EXCEL file describing the information of 108 F1c mtDNAs. Click here for file

Additional file 4 **List of M9a'b complete sequences that were included in Figure **2. This is a DOC file describing the information of 120 complete M9a'b mtDNA genomes and the references for all additional files. Click here for file
