# Supplementary material for: Inland post-glacial dispersal in East Asia revealed by mitochondrial haplogroup M9a'b
Source: BMC Biol. 2011 Jan 10;9:2. doi: 10.1186/1741-7007-9-2 (PMC3027199; doi:10.1186/1741-7007-9-2)
Supplement: Additional file 4 — List of M9a'b complete sequences that were included in Figure 2. This is a DOC file describing the information of 120 complete M9a'b mtDNA genomes and the references for all additional files. [file 1741-7007-9-2-S4.doc]

**Additional file 4 - List of haplogroup M9a’b complete sequences included in Figure 2.**

| **ID^a^** | **Sample** | **Haplogroup** | **Origin** | **References** | **Access No.** |
| --- | --- | --- | --- | --- | --- |
| 1 | VN-Cham 10 | M9b | Cham/Binh Thuan, Vietnam | This study | HM346881 |
| 2 | XJ-Hui 45 | M9b | Hui/Xinjiang | Kong et al. 2006 | DQ272112 |
| 3 | Taiwan SL003 | M9a4a | Taiwan | Soares et al. 2008 | EF093554 |
| 4 | VNM 235 | M9a4a | Vietnam | Soares et al. 2008 | EF185793 |
| 5 | QD-H 8155 | M9a4a | Han/Qingdao, Shandong | This study | HM346882 |
| 6 | VN-Kinh 80 | M9a4a | Kinh/Ha Noi, Vietnam | This study | HM346883 |
| 7 | GX-ML 12 | M9a4a | Mulam/Guangxi | This study | HM346884 |
| 8 | VN-Kinh 41 | M9a4a | Kinh/Hanoi, Vietnam | This study | HM346885 |
| 9 | VN-Kinh 88 | M9a4 | Kinh/Hanoi, Vietnam | This study | HM346886 |
| 10 | HI-Li 145 | M9a5 | Li/Hainan | This study | HM346887 |
| 11 | GX-ML 49 | M9a5 | Mulam/Guangxi | This study | HM346888 |
| 12 | VN-Kinh 53 | M9a5 | Kinh/Hanoi, Vietnam | This study | HM346889 |
| 13 | TH-F34 | M9a5 | Thailand | Joyjinda 2007 | — |
| 14 | TH-C74 | M9a5 | Thailand | Joyjinda 2007 | — |
| 15 | J-SC 0108 | M9a* | Japan | Bilal et al. 2008 | AP010767 |
| 16 | GD-H 7822 | M9a* | Han/Guangdong | This study | HM346890 |
| 17 | GD-H N108 | M9a* | Han/Guangdong | This study | HM346891 |
| 18 | HN-H H27 | M9a1* | Han/Hunan | This study | HM346892 |
| 19^c^ | LN-H 7584 | M9a1b | Han/Liaoning | This study | HM346893 |
| 20 | Naga 514 | M9a1b | Naga/Sagaing, Myanmar | This study | HM346895 |
| 21 | Naga 496 | M9a1b | Naga/Sagaing, Myanmar | This study | HM346896 |
| 22 | YN-T 85 | M9a1b | Tibetan/Yunnan | This study | HM346898 |
| 23 | SC-H D786 | M9a1b | Han/Dujiangyan, Sichuan | This study | HM346899 |
| 24 | GS-T 79 | M9a1b | Tibetan/Gansu | This study | HM346894 |
| 25 | RK-T 339 | M9a1b | Tibetan/Shigatse, Tibet | This study | HM346897 |
| 26 | CD-T 5630 | M9a1b | Tibetan/Chamdo, Tibet | Qin et al. 2010 | GQ895143 |
| 27 | AL-T 2812 | M9a1b1 | Tibetan/Ngari, Tibet | Qin et al. 2010 | GQ895140 |
| 28 | RK-T 412 | M9a1b1 | Tibetan/Shigatse, Tibet | Zhao et al. 2009 | FJ543810 |
| 29 | LS-T 5592 | M9a1b1 | Tibetan/Lhasa, Tibet | Qin et al. 2010 | GQ895148 |
| 30 | CD-T 5980 | M9a1b1 | Tibetan/Chamdo, Tibet | Qin et al. 2010 | GQ895145 |
| 31 | INE 128 | M9a1b1 | Indonesia | Soares et al. 2008 | EF093545 |
| 32 | TH-C112 | M9a1b1 | Thailand | Joyjinda 2007 | — |
| 33 | TH-DM4 | M9a1b1 | Thailand | Luangtrakool 2006 | — |
| 34 | SK 39 | M9a1b1 | Sonowal Kachari/Arunachal | Chandrasekar et al. 2009 | FJ383310 |
| 35 | GL 4 | M9a1b1 | Gallong/Arunachal | Chandrasekar et al. 2009 | FJ383312 |
| 36 | SK 100 | M9a1b1 | Sonowal Kachari/Arunachal | Chandrasekar et al. 2009 | FJ383313 |
| 37 | SK 96 | M9a1b1 | Sonowal Kachari/Arunachal | Chandrasekar et al. 2009 | FJ383318 |
| 38 | SK 78 | M9a1b1 | Sonowal Kachari/Arunachal | Chandrasekar et al. 2009 | FJ383316 |
| 39 | SK 80 | M9a1b1 | Sonowal Kachari/Arunachal | Chandrasekar et al. 2009 | FJ383317 |
| 40 | WA 30 | M9a1b1 | Wanchoo/Arunachal | Chandrasekar et al. 2009 | FJ383322 |
| 41 | GL 26 | M9a1b1 | Gallong/Arunachal | Chandrasekar et al. 2009 | FJ383311 |
| 42 | BN 23/2 | M9a1b1 | Bangladeshi/Bangladesh | This study | GQ337542 |
| 43 | Khasi S8 | M9a1b1 | Khasi/Meghalaya | This study | HM346900 |
| 44 | RK-T 709 | M9a1b1 | Tibetan/Shigatse, Tibet | Zhao et al. 2009 | FJ968774 |
| 45 | SK 72 | M9a1b1 | Sonowal Kachari/Arunachal | Chandrasekar et al. 2009 | FJ383315 |
| 46 | SK 106 | M9a1b1 | Sonowal Kachari/Arunachal | Chandrasekar et al. 2009 | FJ383314 |
| 47 | WA 60 | M9a1b1 | Wanchoo/Arunachal | Chandrasekar et al. 2009 | FJ383323 |
| 48 | WA 106 | M9a1b1 | Wanchoo/Arunachal | Chandrasekar et al. 2009 | FJ383321 |
| 49 | LP 60 | M9a1b1 | Lepcha/Sikkim | Chandrasekar et al. 2009 | FJ383327 |
| 50 | LP 71 | M9a1b1 | Lepcha/Sikkim | Chandrasekar et al. 2009 | FJ383328 |
| 51 | LP 88 | M9a1b1 | Lepcha/Sikkim | Chandrasekar et al. 2009 | FJ383329 |
| 52 | LP 92 | M9a1b1 | Lepcha/Sikkim | Chandrasekar et al. 2009 | FJ383330 |
| 53 | LP105 | M9a1b1 | Lepcha/Sikkim | Chandrasekar et al. 2009 | FJ383326 |
| 54 | Dhm 23 | M9a1a2 | Dhimal/West Bengal | This study | GQ337575 |
| 55 | RK-T 315 | M9a1a2 | Tibetan/Shigatse, Tibet | Zhao et al. 2009 | GU014567 |
| 56 | NQ-T 5621 | M9a1a2 | Tibetan/Nagqu, Tibet | Qin et al. 2010 | GQ895159 |
| 57 | NQ-T 2798 | M9a1a2 | Tibetan/Nagqu, Tibet | Zhao et al. 2009 | FJ968772 |
| 58 | NQ-T 5724 | M9a1a2 | Tibetan/Nagqu, Tibet | Qin et al. 2010 | GQ895160 |
| 59 | YN-T 61 | M9a1a2 | Tibetan/Yunnan | Zhao et al. 2009 | FJ968775 |
| 60 | QH-Tu 39 | M9a1a2 | Tu/Qinghai | This study | HM346901 |
| 61 | Lhob 34 | M9a1a2 | Lhoba/Nyingchi, Tibet | This study | HM346902 |
| 62^e^ | XJ-Kazak 13 | M9a1a* | Kazak/Xinjiang | This study | HM346903 |
| 63 | YN-H 020 | M9a1a* | Han/Yunnan | This study | HM346904 |
| 64 | SN-H 11739 | M9a1a* | Han/Shaanxi | This study | HM346905 |
| 65 | J-SC 0027 | M9a1a1a | Japan | Bilal et al. 2008 | AP010687 |
| 66 | J-ON 0144 | M9a1a1a | Japan | Tanaka et al. 2004 | AP008629 |
| 67 | J-ND 0099 | M9a1a1a | Japan | Tanaka et al. 2004 | AP008766 |
| 68 | J-HN 0226 | M9a1a1a | Japan | Tanaka et al. 2004 | AP008702 |
| 69 | J-HN 0236 | M9a1a1a | Japan | Tanaka et al. 2004 | AP008710 |
| 70 | J-JD 0045 | M9a1a1a | Japan | Tanaka et al. 2004 | AP008863 |
| 71 | J-SC 0002 | M9a1a1a | Japan | Bilal et al. 2008 | AP010662 |
| 72 | J-HN 0190 | M9a1a1a | Japan | Tanaka et al. 2004 | AP008677 |
| 73 | J-ND 0207 | M9a1a1a | Japan | Tanaka et al. 2004 | AP008815 |
| 74 | J-SP 0053 | M9a1a1a | Japan | Ueno et al. 2009 | AP011019 |
| 75 | Nivkchi 149 | M9a1a1a | Nivikchi/North Asia | Ingman, Gyllensten 2007 | EU007852 |
| 76 | Chi 3634 | M9a1a1a | Chinese/China | Ingman et al. 2000 | AF346972 |
| 77 | HA-H C133 | M9a1a1a | Han/Henan | This study | HM346906 |
| 78 | HA-H 1418 | M9a1a1* | Han/Henan | This study | HM346907 |
| 79 | J-JD 0041 | M9a1a1b | Japan | Tanaka et al. 2004 | AP008860 |
| 80 | J-PD 0043 | M9a1a1b | Japan | Tanaka et al. 2004 | AP008378 |
| 81 | IM-M 239 | M9a1a1b | Mongolian/Inner Mongolia | This study | HM346908 |
| 82 | SC-H B5382 | M9a1a1* | Han/Yanting, Sichuan | This study | HM346909 |
| 83 | YN-B 11 | M9a1a1* | Bai/Yunnan | This study | HM346910 |
| 84 | QH-Salar 3 | M9a1a1* | Salar/Qinghai | This study | HM346911 |
| 85 | GD-H N227 | M9a1a1* | Han/Shantou, Guangdong | This study | HM346912 |
| 86 | XJ-Hui 42 | M9a1a1c | Hui/Xinjiang | This study | HM346913 |
| 87 | HA-H 12123 | M9a1a1c1 | Han/Henan | This study | HM346914 |
| 88 | SC-H 1893 | M9a1a1c1b | Han/Dujiangyan, Sichuan | This study | HM346915 |
| 89 | DR 46 | M9a1a1c1b | Dirang Monpa/Arunachal | Chandrasekar et al. 2009 | FJ383325 |
| 90 | DR 100 | M9a1a1c1b | Dirang Monpa/Arunachal | Chandrasekar et al. 2009 | FJ383324 |
| 91 | LAC 70 | M9a1a1c1b | Lachungpa/Sikkim | Chandrasekar et al. 2009 | FJ383320 |
| 92 | LAC 32 | M9a1a1c1b | Lachungpa/Sikkim | Chandrasekar et al. 2009 | FJ383319 |
| 93 | Lach 21 | M9a1a1c1b | Lachungpa/Sikkim | This study | GQ337588 |
| 94 | LS-T 5629 | M9a1a1c1b | Tibetan/Lhasa, Tibet | Qin et al. 2010 | GQ895150 |
| 95 | LS-T 5632 | M9a1a1c1b | Tibetan/Lhasa, Tibet | Qin et al. 2010 | GQ895151 |
| 96 | LS-T 7 | M9a1a1c1b | Tibetan/Lhasa, Tibet | Qin et al. 2010 | GQ895146 |
| 97 | NQ-T 2827 | M9a1a1c1b | Tibetan/Nagqu, Tibet | This study | HM346918 |
| 98 | RK-T 733 | M9a1a1c1b | Tibetan/Shigatse, Tibet | This study | HM346916 |
| 99 | RK-T 1139 | M9a1a1c1b | Tibetan/Shigatse, Tibet | This study | HM346917 |
| 100 | NQ-T 2759 | M9a1a1c1b | Tibetan/Nagqu, Tibet | This study | HM346919 |
| 101 | NQ-T 3890 | M9a1a1c1b | Tibetan/Nagqu, Tibet | This study | HM346920 |
| 102 | NQ-T 3780 | M9a1a1c1b | Tibetan/Nagqu, Tibet | This study | HM346921 |
| 103 | RK-T 528 | M9a1a1c1b | Tibetan/Shigatse, Tibet | This study | HM346922 |
| 104 | NQ-T 2767 | M9a1a1c1b | Tibetan/Nagqu, Tibet | This study | HM346923 |
| 105 | Monp 22 | M9a1a1c1b | Monpa/Nyingchi, Tibet | This study | HM346924 |
| 106 | Monp 25 | M9a1a1c1b | Monpa/Nyingchi, Tibet | This study | HM346925 |
| 107 | NQ-T 2790 | M9a1a1c1b | Tibetan/Nagqu, Tibet | This study | HM346926 |
| 108 | NQ-T 2808 | M9a1a1c1b | Tibetan/Nagqu, Tibet | This study | HM346927 |
| 109 | RK-T 939 | M9a1a1c1b | Tibetan/Shigatse, Tibet | This study | HM346928 |
| 110 | IM-Br 36 | M9a1a1c1b | Buryat/Inner Mongolia | This study | HM346929 |
| 111 | YN-T 70 | M9a1a1c1b | Tibetan/Yunnan | This study | HM346930 |
| 112 | SC-T 011 | M9a1a1c1b | Tibetan/Liangshan, Sichuan | This study | HM346931 |
| 113 | SD-H 10327 | M9a1a1c1a | Han/Tai'an, Shandong | This study | HM346932 |
| 114 | QD-H 8125 | M9a1a1c1a | Han/Qingdao, Shandong | This study | HM346933 |
| 115 | LN-H 7606 | M9a1a1c1a | Han/Liaoning | This study | HM346934 |
| 116 | IM-M 223 | M9a1a1c1a | Mongolian/Inner Mongolia | This study | HM346935 |
| 117 | SC-T 030 | M9a1a1c1a | Tibetan/Liangshan, Sichuan | This study | HM346936 |
| 118 | XJ-H 8420 | M9a1a1c1a | Han/Xinjiang | Kong et al. 2003b | AY255153 |
| 119 | J-HN 0228 | M9a1a1c1a | Japan | Tanaka et al. 2004 | AP008704 |
| 120 | J-PD 0011 | M9a1a1c1a | Japan | Tanaka et al. 2004 | AP008353 |

^a^ ID numbers correspond to the numbers in Figure 2.

**Supplementary References**

Allard, MW, MR Wilson, KL Monson, B Budowle. 2004. Control region sequences for East Asian individuals in the Scientific Working Group on DNA Analysis Methods forensic mtDNA data set. Leg Med (Tokyo) 6:11-24.

Asari, M, K Umetsu, N Adachi, J Azumi, K Shimizu, H Shiono. 2007. Utility of haplogroup determination for forensic mtDNA analysis in the Japanese population. Leg Med (Tokyo) 9:237-240.

Bamshad, MJ, WS Watkins, ME Dixon, LB Jorde, BB Rao, JM Naidu, BVR Prasad, A Rasanayagam, MF Hammer. 1998. Female gene flow stratifies Hindu castes. Nature 395:651-652.

Bilal, E, R Rabadan, G Alexe, et al. 2008. Mitochondrial DNA haplogroup D4a is a marker for extreme longevity in Japan. PLoS ONE 3:e2421.

Black, ML, K Dufall, C Wise, S Sulliva, AH Bittles. 2006. Genetic ancestries in northwest Cambodia. Ann Hum Biol 33:620-627.

Chandrasekar, A, S Kumar, J Sreenath, et al. 2009. Updating phylogeny of mitochondrial DNA macrohaplogroup M in India: dispersal of modern human in South Asian corridor. PLoS ONE 4:e7447.

Chen, F, Y Deng, Y Dang, B Zhang, H Mu, X Yu, L Li, C Yan, T Chen. 2008a. Genetic polymorphism of mitochondrial DNA HVS-I and HVS-II of Chinese Tu ethnic minority group J Genet Genomics 35:225-232.

Chen, F, SY Wang, RZ Zhang, YH Hu, GF Gao, YH Liu, QP Kong. 2008b. Analysis of mitochondrial DNA polymorphisms in Guangdong Han Chinese. Forensic Sci Int Genet 2:150-153.

Cheng, BW, WR Tang, L He, YL Dong, J Lu, YP Lei, HJ Yu, JL Zhang, CJ Xiao. 2008. Genetic imprint of the Mongol: signal from phylogeographic analysis of mitochondrial DNA. J Hum Genet 53:905-913.

Comas, D, F Calafell, E Mateu, et al. 1998. Trading genes along the silk road: mtDNA sequences and the origin of central Asian populations. Am J Hum Genet 63:1824-1838.

Comas, D, S Plaza, RS Wells, N Yuldaseva, O Lao, F Calafell, J Bertranpetit. 2004. Admixture, migrations, and dispersals in Central Asia: evidence from maternal DNA lineages. Eur J Hum Genet 12:495-504.

Cordaux, R, N Saha, GR Bentley, R Aunger, SM Sirajuddin, M Stoneking. 2003. Mitochondrial DNA analysis reveals diverse histories of tribal populations from India. Eur J Hum Genet 11:253-264.

Derenko, M, B Malyarchuk, T Grzybowski, et al. 2007. Phylogeographic analysis of mitochondrial DNA in northern Asian Populations. Am J Hum Genet 81:1025-1041.

Derenko, MV, T Grzybowski, BA Malyarchuk, et al. 2003. Diversity of mitochondrial DNA lineages in South Siberia. Ann Hum Genet 67:391-411.

Fornarino, S, M Pala, V Battaglia, R Maranta, A Achilli, G Modiano, A Torroni, O Semino, SA Santachiara-Benerecetti. 2009. Mitochondrial and Y-chromosome diversity of the Tharus (Nepal): a reservoir of genetic variation. BMC Evol Biol 9:154.

Fucharoen, G, S Fucharoen, S Horai. 2001. Mitochondrial DNA polymorphisms in Thailand. J Hum Genet 46:115-125.

Gan, RJ, SL Pan, LF Mustavich, et al. 2008. Pinghua population as an exception of Han Chinese's coherent genetic structure. J Hum Genet 53:303-313.

Horai, S, K Murayama, K Hayasaka, S Matsubayashi, Y Hattori, G Fucharoen, S Harihara, KS Park, K Omoto, IH Pan. 1996. MtDNA polymorphism in East Asian populations, with special reference to the peopling of Japan. Am J Hum Genet 59:579-590.

Imaizumi, K, TJ Parsons, M Yoshino, MM Holland. 2002. A new database of mitochondrial DNA hypervariable regions I and II sequences from 162 Japanese individuals. Int J Legal Med 116:68-73.

Ingman, M, U Gyllensten. 2007. Rate variation between mitochondrial domains and adaptive evolution in humans. Hum Mol Genet 16:2281-2287.

Ingman, M, H Kaessmann, S Paabo, U Gyllensten. 2000. Mitochondrial genome variation and the origin of modern humans. Nature 408:708-713.

Irwin, JA, A Ikramov, J Saunier, et al. 2010. The mtDNA composition of Uzbekistan: a microcosm of Central Asian patterns. Int J Legal Med 124:195-204.

Irwin, JA, JL Saunier, P Beh, KM Strouss, CD Paintner, TJ Parsons. 2009. Mitochondrial DNA control region variation in a population sample from Hong Kong, China. Forensic Sci Int Genet 3:e119-125.

Irwin, JA, JL Saunier, KM Strouss, TM Diegoli, KA Sturk, JE O'Callaghan, CD Paintner, C Hohoff, B Brinkmann, TJ Parsons. 2008. Mitochondrial control region sequences from a Vietnamese population sample. Int J Legal Med 122:257-259.

Ji, Y, AM Zhang, X Jia, YP Zhang, X Xiao, S Li, X Guo, HJ Bandelt, Q Zhang, YG Yao. 2008. Mitochondrial DNA haplogroups M7b1'2 and M8a affect clinical expression of leber hereditary optic neuropathy in Chinese families with the m.11778G-->a mutation. Am J Hum Genet 83:760-768.

Jin, H-J, C Tyler-Smith, W Kim. 2009. The peopling of Korea revealed by analyses of mitochondrial DNA and Y-chromosomal markers. PLoS ONE 4:e4210.

Jin, HJ, KD Kwak, SB Hong, DJ Shin, MS Han, C Tyler-Smith, W Kim. 2006. Forensic genetic analysis of mitochondrial DNA hypervariable region I/II sequences: an expanded Korean population database. Forensic Sci Int 158:125-130.

Joyjinda, Y. 2007. The role of mitochondrial background in the expression of leber hereditary optic neuropathy (LHON): Mahidol University.

Keyser-Tracqui, C, E Crubezy, H Pamzsav, T Varga, B Ludes. 2006. Population origins in Mongolia: Genetic structure analysis of ancient and modern DNA. Am J Phys Anthropol 131:272-281.

Kivisild, T, MJ Bamshad, K Kaldma, et al. 1999. Deep common ancestry of Indian and western-Eurasian mitochondrial DNA lineages. Curr Biol 9:1331-1334.

Kivisild, T, S Rootsi, M Metspalu, et al. 2003. The genetic heritage of the earliest settlers persists both in Indian tribal and caste populations. Am J Hum Genet 72:313-332.

Kivisild, T, HV Tolk, J Parik, YM Wang, SS Papiha, HJ Bandelt, R Villems. 2002. The emerging limbs and twigs of the East Asian mtDNA tree. Mol Biol Evol 19:1737-1751.

Kolman, CJ, N Sambuughin, E Bermingham. 1996. Mitochondrial DNA analysis of mongolian populations and implications for the origin of New World founders. Genetics 142:1321-1334.

Kong, QP, HJ Bandelt, C Sun, et al. 2006. Updating the East Asian mtDNA phylogeny: a prerequisite for the identification of pathogenic mutations. Hum Mol Genet 15:2076-2086.

Kong, QP, YG Yao, M Liu, SP Shen, C Chen, CL Zhu, MG Palanichamy, YP Zhang. 2003a. Mitochondrial DNA sequence polymorphisms of five ethnic populations from northern China. Hum Genet 113:391-405.

Kong, QP, YG Yao, C Sun, HJ Bandelt, CL Zhu, YP Zhang. 2003b. Phylogeny of East Asian mitochondrial DNA lineages inferred from complete sequences. Am J Hum Genet 73:671-676.

Koyama, H, M Iwasa, Y Maeno, T Tsuchimochi, I Isobe, Y Seko-Nakamura, J Monma-Ohtaki, T Matsumoto, S Ogawa. 2002. Mitochondrial sequence haplotype in the Japanese population. Forensic Sci Int 125:93-96.

Lee, H, JE Yoo, M Park, U Chung, KJ Shin. 2006a. Mitochondrial DNA control region sequences in Koreans: identification of useful variable sites and phylogenetic analysis for mtDNA data quality control. Int J Legal Med 120:5-14.

Lee, HY, JE Yoo, MJ Park, U Chung, CY Kim, KJ Shin. 2006b. East Asian mtDNA haplogroup determination in Koreans: haplogroup-level coding region SNP analysis and subhaplogroup-level control region sequence analysis. Electrophoresis 27:4408-4418.

Lee, SD, YS Lee, JB Lee. 2002. Polymorphism in the mitochondrial cytochrome B gene in Koreans - an additional marker for individual identification. Int J Legal Med 116:74-78.

Lertrit, P, S Poolsuwan, R Thosarat, T Sanpachudayan, H Boonyarit, C Chinpaisal, B Suktitipat. 2008. Genetic history of Southeast Asian populations as revealed by ancient and modern human mitochondrial DNA analysis. Am J Phys Anthropol 137:425-440.

Li, B, F Zhong, H Yi, X Wang, L Li, L Wang, X Qi, L Wu. 2007a. Genetic polymorphism of mitochondrial DNA in Dong, Gelao, Tujia, and Yi ethnic populations from Guizhou, China. J Genet Genomics 34:800-810.

Li, H, XY Cai, ER Winograd-Cort, et al. 2007b. Mitochondrial DNA diversity and population differentiation in Southern East Asia. Am J Phys Anthropol 134:481-488.

Liu, C, SY Wang, M Zhao, ZY Xu, YH Hu, F Chen, RZ Zhang, GF Gao, YS Yu, QP Kong. 2010. Mitochondrial DNA polymorphisms in Gelao ethnic group residing in Southwest China. Forensic Sci Int Genet 20:20.

Liu, XS, T Chen, SB Li. 2004. Sequence polymorphism of human mitochondrial DNA control region in Chinese Dongxiang unrelated individuals. Journal of Medical Colleges of PLA 19:259-262.

Liu, XS, SB Li. 2003. Polymorphism of mitochondrial DNA D-loop region in Chinese BAOAN ethnic group. Journal of The Fourth Military Medical University 24:1832-1836.

Liu, XS, SB Li. 2004a. Mitochondrial DNA Polymorphsim in Control Region from Chinese Yugu Population. Academic Journal of Xi an Jiaotong University 15:174-177.

Liu, XS, SB Li. 2004b. Study on polymorphisms of mitochondrial DNA D-loop region in the Sala population in China. Journal of Xi'an Jiaotong University (Medical Sciences) 25:213-216.

Luangtrakool, K. 2006. Mitochondrial genomes and diabetes mellitus type 2 in Thailand: Mahidol University.

Mabuchi, T, R Susukida, A Kido, M Oya. 2007. Typing the 1.1 kb control region of human mitochondrial DNA in Japanese individuals. J Forensic Sci 52:355-363.

Maruyama, S, K Minaguchi, N Saitou. 2003. Sequence polymorphisms of the mitochondrial DNA control region and phylogenetic analysis of mtDNA lineages in the Japanese population. Int J Legal Med 117:218-225.

Melton, T, S Clifford, E Martinson, M Batzer, M Stoneking. 1998. Genetic evidence for the proto-Austronesian homeland in Asia: mtDNA and nuclear DNA variation in Taiwanese aboriginal tribes. Am J Hum Genet 63:1807-1823.

Metspalu, M, T Kivisild, E Metspalu, et al. 2004. Most of the extant mtDNA boundaries in South and Southwest Asia were likely shaped during the initial settlement of Eurasia by anatomically modern humans. BMC Genet 5.

Mountain, JL, JM Hebert, S Bhattacharyya, PA Underhill, C Ottolenghi, M Gadgil, LL Cavallisforza. 1995. Demographic history of India and mtDNA-sequence diversity. Am J Hum Genet 56:979-992.

Nagai, A, I Nakamura, F Shiraki, Y Bunai, I Ohya. 2003. Sequence polymorphism of mitochondrial DNA in Japanese individuals from Gifu Prefecture. Leg Med (Tokyo) 5 Suppl 1:S210-213.

Nishimaki, Y, K Sato, L Fang, M Ma, H Hasekura, B Boettcher. 1999. Sequence polymorphism in the mtDNA HV1 region in Japanese and Chinese. Leg Med (Tokyo) 1:238-249.

Oota, H, T Kitano, F Jin, I Yuasa, L Wang, S Ueda, N Saitou, M Stoneking. 2002. Extreme mtDNA homogeneity in continental Asian populations. Am J Phys Anthropol 118:146-153.

Oota, H, W Settheetham-Ishida, D Tiwawech, T Ishida, M Stoneking. 2001. Human mtDNA and Y-chromosome variation is correlated with matrilocal versus patrilocal residence. Nat Genet 29:20-21.

Peng, MS, HH Quang, KP Dang, AV Trieu, HW Wang, YG Yao, QP Kong, YP Zhang. 2010. Tracing the Austronesian footprint in Mainland Southeast Asia: a perspective from mitochondrial DNA. Mol Biol Evol 27:2417-2430.

Pfeiffer, H, R Steighner, R Fisher, H Mornstad, CL Yoon, MM Holland. 1998. Mitochondrial DNA extraction and typing from isolated dentin-experimental evaluation in a Korean population. Int J Legal Med 111:309-313.

Powell, GT, HM Yang, C Tyler-Smith, YL Xue. 2007. The population history of the Xibe in northern China: A comparison of autosomal, mtDNA and Y-chromosomal analyses of migration and gene flow. Forensic Sci Int Genet 1:115-119.

Qian, YP, ZT Chu, Q Dai, CD Wei, JY Chu, A Tajima, S Horai. 2001. Mitochondrial DNA polymorphisms in Yunnan nationalities in China. J Hum Genet 46:211-220.

Qin, Z, Y Yang, L Kang, et al. 2010. A mitochondrial revelation of early human migrations to the Tibetan Plateau before and after the last glacial maximum. Am J Phys Anthropol 143:555-569.

Quintana-Murci, L, R Chaix, RS Wells, et al. 2004. Where west meets east: the complex mtDNA landscape of the southwest and Central Asian corridor. Am J Hum Genet 74:827-845.

Reddy, BM, BT Langstieh, V Kumar, T Nagaraja, ANS Reddy, A Meka, AG Reddy, K Thangaraj, L Singh. 2007. Austro-Asiatic tribes of Northeast India provide hitherto missing genetic link between South and Southeast Asia. PLoS ONE 2:e1141.

Roychoudhury, S, S Roy, A Basu, R Banerjee, H Vishwanathan, MVU Rani, SK Sil, M Mitra, PP Majumder. 2001. Genomic structures and population histories of linguistically distinct tribal groups of India. Hum Genet 109:339-350.

Seo, Y, B Stradmann-Bellinghausen, C Rittner, K Takahama, PM Schneider. 1998. Sequence polymorphism of mitochondrial DNA control region in Japanese. Forensic Sci Int 97:155-164.

Soares, P, JA Trejaut, JH Loo, et al. 2008. Climate change and postglacial human dispersals in Southeast Asia. Mol Biol Evol 25:1209-1218.

Sykes, B, A Leiboff, J Lowbeer, S Tetzner, M Richards. 1995. The origins of the Polynesians - an interpretation from mitochondrial lineage analysis. Am J Hum Genet 57:1463-1475.

Tajima, A, M Hayami, K Tokunaga, T Juji, M Matsuo, S Marzuki, K Omoto, S Horai. 2004. Genetic origins of the Ainu inferred from combined DNA analyses of maternal and paternal lineages. J Hum Genet 49:187-193.

Tanaka, M, VM Cabrera, AM González, et al. 2004. Mitochondrial genome variation in Eastern Asia and the peopling of Japan. Genome Res 14:1832-1850.

Thangaraj, K, GV Ramana, L Singh. 1999. Y-chromosome and mitochondrial DNA polymorphisms in Indian populations. Electrophoresis 20:1743-1747.

Trejaut, JA, T Kivisild, JH Loo, CL Lee, CL He, CJ Hsu, ZY Li, M Lin. 2005. Traces of archaic mitochondrial lineages persist in Austronesian-speaking Formosan populations. PLoS Biol 3:1362-1372.

Tsai, LC, CY Lin, JCI Lee, JG Chang, A Linacre, W Goodwin. 2001. Sequence polymorphism of mitochondrial D-loop DNA in the Taiwanese Han population. Forensic Sci Int 119:239-247.

Ueno, H, Y Nishigaki, Q-P Kong, N Fuku, S Kojima, N Iwata, N Ozaki, M Tanaka. 2009. Analysis of mitochondrial DNA variants in Japanese patients with schizophrenia. Mitochondrion 9:385-393.

Wang, WZ, CY Wang, YT Cheng, AL Xu, CL Zhu, SF Wu, QP Kong, YP Zhang. 2010. Tracing the origins of Hakka and Chaoshanese by mitochondrial DNA analysis. Am J Phys Anthropol 141:124-130.

Wen, B, H Li, S Gao, et al. 2005. Genetic structure of Hmong-Mien speaking populations in East Asia as revealed by mtDNA lineages. Mol Biol Evol 22:725-734.

Wen, B, H Li, DR Lu, et al. 2004a. Genetic evidence supports demic diffusion of Han culture. Nature 431:302-305.

Wen, B, XH Xie, S Gao, et al. 2004b. Analyses of genetic structure of Tibeto-Burman populations reveals sex-biased admixture in southern Tibeto-Burmans. Am J Hum Genet 74:856-865.

Yao, YG, QP Kong, HJ Bandelt, T Kivisild, YP Zhang. 2002a. Phylogeographic differentiation of mitochondrial DNA in Han Chinese. Am J Hum Genet 70:635-651.

Yao, YG, QP Kong, XY Man, HJ Bandelt, YP Zhang. 2003. Reconstructing the evolutionary history of China: a caveat about inferences drawn from ancient DNA. Mol Biol Evol 20:214-219.

Yao, YG, QP Kong, CY Wang, CL Zhu, YP Zhang. 2004. Different matrilineal contributions to genetic structure of ethnic groups in the Silk Road region in China. Mol Biol Evol 21:2265-2280.

Yao, YG, L Nie, H Harpending, YX Fu, ZG Yuan, YP Zhang. 2002b. Genetic relationship of Chinese ethnic populations revealed by mtDNA sequence diversity. Am J Phys Anthropol 118:63-76.

Yao, YG, YP Zhang. 2002. Phylogeographic analysis of mtDNA variation in four ethnic populations from Yunnan Province: new data and a reappraisal. J Hum Genet 47:311-318.

Zhao, M, QP Kong, HW Wang, et al. 2009. Mitochondrial genome evidence reveals successful Late Paleolithic settlement on the Tibetan Plateau. Proc Natl Acad Sci U S A 106:21230-21235.

Zimmermann, B, M Bodner, S Amory, L Fendt, A Rock, D Horst, B Horst, T Sanguansermsri, W Parson, A Brandstatter. 2009. Forensic and phylogeographic characterization of mtDNA lineages from northern Thailand (Chiang Mai). Int J Legal Med 123:495-501.
